# Comparative study of the tibial tubercle-trochlear groove distance measured in two ways and tibial tubercle-posterior cruciate ligament distance in patients with patellofemoral instability

**DOI:** 10.1186/s13018-020-01726-2

**Published:** 2020-06-08

**Authors:** Lei Shu, Qubo Ni, Xu Yang, Biao Chen, Hua Wang, Liaobin Chen

**Affiliations:** Department of Orthopedic Surgery, Zhongnan Hospital of Wuhan University, Wuhan, 430071 China

**Keywords:** Patellofemoral instability, Patella dislocation, TT-TG distance, TT-TG distance under three-dimensional CT reconstruction, TT-PCL distance

## Abstract

**Background:**

A comparative analysis of the strengths and weaknesses of three different methods for radiologic evaluation of patellofemoral instability (PFI).

**Methods:**

Computed tomography (CT) and magnetic resonance imaging (MRI) were performed in 47 patients with or without PFI. The tibial tubercle-trochlear groove (TT-TG) distance was measured by two observers through conventional CT and three-dimensional CT reconstruction (TDR-TT-TG) respectively and the tibial tubercle-posterior cruciate ligament (TT-PCL) distance with MRI. The intraclass correlation coefficient (ICC) was used to evaluate the interobserver reliability. In addition, the differences of three measurements between different patients were compared. The consistency of TT-TG and TDR-TT-TG was analyzed by the Bland-Altman method.

**Results:**

The ICCs of three measurements were high between two observers; the results were TT-TG (ICC = 0.852), TDR-TT-TG (ICC = 0.864), and TT-PCL (ICC = 0.758). The values of PFI patients were significantly higher than those of non-PFI patients, and the mean TT-TG, TDR-TT-TG, and TT-PCL distance in patients with PFI were 19.0 ± 3.8 mm, 19.0 ± 3.7 mm, and 25.1 ± 3.6 mm, respectively. There was no statistically significant difference between the TT-TG distance and the TDR-TT-TG distance, we found no significant difference. The Bland-Altman analysis showed that the TDR-TT-TG distance was in good agreement with the TT-TG distance.

**Conclusion:**

All three methods can be used to assess PFI; the TDR-TT-TG measurement method has superior operability and better interobserver consistency. It may be an alternative method to the conventional TT-TG distance measurement.

## Introduction

Patellofemoral instability (PFI) refers to the fact that the patella cannot slide normally in the femoral trochlear groove, causing one or more subluxations or dislocations and abnormal patella slippage, resulting in a series of symptoms [1]. PFI is a common disease in orthopedic sports medicine [2], especially in women aged 10–17 years [3–5]. The incidence rate is approximately between 7 and 49 people per 100,000 [3, 5, 6]. If the patellofemoral joint is unstable, every instance of flexion and extension of the knee joint will cause uneven stress and wear of the articular cartilage. Long-term instability can lead to serious consequences such as cartilage injury, patellofemoral joint degeneration, and quadriceps atrophy [7–9]. Although the reversed dynamic patellar apprehension test was recently introduced by Zimmermann et al. [10] as a reliable clinical examination tool for the diagnosis of PFI, but its clinical use still needs more research confirmation. At present, the diagnosis of PFI still mainly relies on the comprehensive judgment of combining the patients’ medical history with a detailed physical examination and imaging procedures.

After the failure of conservative treatment for PFI, surgery is frequently needed. To date, the operation for an unstable patella is mainly based on the principle of the balancing of soft tissue and bone structure [4, 11, 12]. Bone repair procedures mainly include tibial internal osteotomy and femoral trochleoplasty [13, 14]. Whether the distal realignment procedure is performed, the distance of tibial tubercle-trochlear groove (TT-TG) is often applied [4]. This radiographic parameter was originally described by Goutallier et al. [13] in 1978 on an axial radiograph. Dejour et al. [15] first used the computed tomography (CT) technology to measure the TT-TG distance, which is considered to be an important reference for quantifying PFI. In addition, magnetic resonance imaging (MRI) has been gradually used to identify the position of the tibial tuberosity, with the advantages of evaluating soft tissue structures and the articular cartilage with no radiation [16–18]. However, the TT-TG distance measured on CT images is still considered as a gold standard [6, 19–21]. Several studies have emphasized factors that may have an effect on the value of TT-TG distance, such as trochlear dysplasia and knee rotation [16, 22, 23].To expel the influence of these variables, Seitlinger et al. [19, 23, 24] have proposed to judge PFI with MRI—the tibial tubercle-posterior cruciate ligament (TT-PCL) distance. The authors believed that the TT-PCL distance is more accurate than the TT-TG distance for identifying the position of the tibial tuberosity, and that is may be an alternative modality for assessing PFI.

**Fig. 1:**
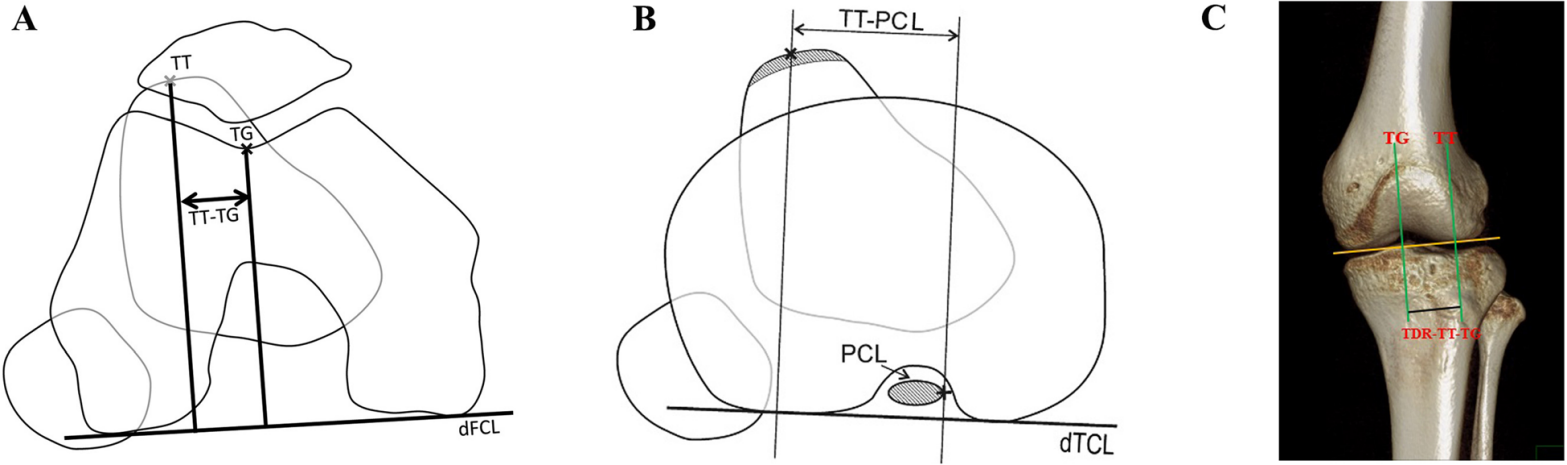
Three different radiologic methods for assessment of the tibial tuberosity position in relationship to the trochlea groove and posterior cruciate ligament respectively**. a** TT-TG, tibial tubercle–trochlear groove distance (performed by CT). **b** TT-PCL, tibial tubercle–posterior cruciate ligament distance (performed by MRI). **c** TDR-TT-TG, TT-TG distance under three-dimensional reconstruction (performed by 3D-CT reconstruction). TT, tibial tuberosity; TG, trochlear groove; dFCL, dorsal femoral condylar line; dTCL, dorsal tibia condylar line

As we all know, the traditional measurement of TT-TG requires overlapping images of two planes, and the TT-PCL measurement requires three layers of superposition, identifying points containing the insertion point of PCL, trochlear groove, tibial tubercle, etc., whereas these markers are not in an axial plane [25, 26] on conventional CT or MRI, which may lead to a low intra- and interobserver reliability to measure the distances. Therefore, to reduce the measurement bias of the former techniques, we projected that PFI can be assessed by measuring the distance between the tibial tuberosity and the femoral trochlear groove with three-dimensional CT reconstruction (TDR) images. This TT-TG distance measured by use of 3D reconstruction is abbreviated (TDR-TT-TG). This method only requires one image to complete the measurement and has the advantage of being simple to carry out (Fig. 1c). The aim of our study was to measure the TT-TG distance by TDR in patients with or without PFI and, furthermore, to compare the measurement consistency of TT-TG distance, TDR-TT-TG distance, and TT-PCL distance.

## Materials and methods

Forty-seven patients with PFI were admitted to the observation group from April 2017 to April 2019 in our hospital. Selection criteria were as follows: (1) clinical diagnosis of PFI and (2) no history of knee surgery. Exclusion criteria were as follows: (1) patients with MRI or CT contraindications; (2) multiple ligament injuries, especially in patients with posterior cruciate ligament injury; (3) patients with knee deformity or severe osteoarthritis; and (4) patients with periarticular fractures. Forty-seven non-PFI patients (non-acute stage gout patients) who underwent CT and MRI examinations on the ipsilateral knee in our hospital were selected as the control group.

### Imaging acquisition

CT and MRI were performed in patients with PFI, and all gout patients underwent MRI and dual-source dual-energy CT examinations. Imaging operations were completed by unified trained technicians, and related parameters used in the scanning of each patient were uniformed. The specific procedures are as follows:

#### CT scan

Conventional CT scan was performed on the Siemens 64-slice CT (Siemens Definition, Erlangen, Germany). All patients were supine with leg and thigh in full extension. Straps were attached on the lower leg to avoid rotation. Images were taken with the following parameters: slice thickness 4 mm, scan time 9.28 s (nearly 5 to 10 s), rotation time 1 s, care dose (120 kV,100 mAs), pitch 0.8 mm, and matrix 512 × 512.

The dual-source dual-energy computed tomography (DSDECT) scan completed on the same instrument (Siemens Definition, Erlangen, Germany). The parameters were as follows: slice thickness 2 mm, scan time 17.14 s (nearly 10 to 20 s), rotation time 1.0 s, care dose (tube A 140 kV, 55 mAs; tube B 80 kV, 55 mAs), pitch 0.7 mm, and matrix 512 × 512.

The conventional CT image was exported with Philips IntelliSpace Portal for transforming into TDR image, the bone window was selected for reconstruction image of the knee joint, and the patella was removed by excluding the Freehand function in clip&3D-segmentation, adjusted to the coronal position. Finally, a TDR image of the knee joint was obtained.

#### MRI scan

All about MRI (Philips MR Systems Ingenia 3.0 T, Andover, Massachusetts) scanner was routine: the patients were in a supine position, with a standard knee coil center level against the lower edge of the patella. The knee and hip joint naturally extended, and the feet were braced to prevent any movement. Four MRI sequences of knee were obtained. The scanning parameters of Andover are given below: (1) coronal proton density-weighted spectral attenuated inversion recovery (PDW-SPAIR) MR images [repetition time millisecond (TR)/echo time millisecond (TE) 1940/30, field of view (FOV) 220 mm × 179 mm, matrix 368 × 245, slice thickness 3 mm, sections per slab 21]; (2) transverse PDW-SPAIR MR images (TR/TE 2036/30, FOV 169 mm × 189 mm, matrix 344 × 264, slice thickness 4 mm, act slice gap 0.4 mm, sections per slab 24); (3) sagittal T1-weighted aTSE (turbo spin-echo) MR images (TR/TE 694/12, FOV 160 mm × 160 mm, matrix 308 × 240, slice thickness 3 mm, act slice gap 0.3 mm, sections per slab 24); and (4) sagittal proton density-weighted spectral inversion recovery (PDW-SPIR) MR images (TR/TE 1,554/30, FOV 160 × 160 mm, matrix 292 × 231, slice thickness 3 mm, act slice gap 0.3 mm, sections per slab 24).

### Measurement

#### TT-TG measurement

As previously mentioned, the TT-TG distance was only measured on CT images according to the method described by Dejour et al. [15]. Two special CT layers were chosen: the first was the level of the “Roman Arch” at the top of the femoral condyle, at the deepest point of the femoral trochlea (TG), and the second layer was the insertion center of the patellar tendon to the tibial tuberosity (TT). The TT-TG distance was calculated by superimposing two layers; TT line and TG line were perpendicular to the tangent line of the dorsal femoral condylar line (dFCL). The TT-TG distance was the distance between the two parallel lines, and the measurement accuracy was 0.1 mm (Fig. 1a).

#### TT-PCL measurement

The measurement was only performed on MRI images according to the TT-PCL distance described by Seitlinger et al. [23]. Three special scanning planes were chosen on the knee joint axial image. The first plane was the dorsal condylar line of the tibia, that was defined just below the articular surface of the tibia plateau and above the fibular head. The second plane was the medial border of the PCL, the plane was defined in the most inferior slice in which the ligament could still be clearly identified, which corresponds with the insertion of the ligament at the tibia. The third slice was the reference point of the tibial tubercle, which was located by marking the midpoint of the insertion of the patellar tendon, then the deepest image which posterior cruciate ligament (PCL) originates on the tibial plateau was selected and marking the inner edge of the PCL, ultimately, choosing a slice that the patellar ligament was completely exposed and marking the center point of the patellar ligament. All planes described above were superimposed by software to obtain a new image. Two perpendicular lines were taken through the inner edge of the PCL and the midpoint of the patellar ligament. The distance between the two parallel lines was the TT-PCL distance (Fig. 1b).

#### TDR-TT-TG measurement

The TDR-TT-TG distance was measured under TDR. First, the tangential line was taken from the two lowest points of the femoral condyle, and then draw two lines through the deepest point of the femoral trochlea (TG) and the most cephalic point of the tibial tuberosity (TT) respectively. Both TT line and TG line were perpendicular to the tangent of the distal femoral condyle. The horizontal distance between the two lines was measured, which was the TDR-TT-TG distance, with a measurement accuracy of 0.1 mm (Fig. 1c).

### Quality control

Both observers were joint and sports doctors with more than 3 years of clinical experience and were blinded to the sex, age, the clinical diagnosis, the observation grouping, and the comparisons of individuals. All measurements were performed independently at the same time, and the intraclass correlation coefficient (ICC) was used to assess interobserver reliability to minimize the bias and confounding factors of measurement.

### Data extraction and analysis

All relevant data were analyzed by SPSS 22.0 (IBM Corp., released 2013, IBM SPSS Statistics for Windows, Armonk, NY: IBM Corp). Continuous variables are expressed as the mean ± standard deviation. The comparison between two groups was conducted with an independent-samples *T* test and the Bland-Altman method for correlation analysis [Bland-Altman scatter plot was drawn by MedCalc v12.1.3 (MedCalc Software bvba, Ostend, Belgium)]. *P* < 0.05 was considered statistically significant.

## Results

### Basic characteristics of the included patients

The PFI group included 19 males and 28 females, aged 11–49 years old, with an average age of 20.7 ± 8.0 years; in the control group, 33 males and 14 females were included in the analysis, aged 23–56 years old, with a mean age of 31.8 ± 12.7 years old.

### Interobserver differences in three measurements assessing PFI patients

The ICCs of the three different measurement methods between the two observers are shown in Table 1. The interobserver agreements between the TT-TG measurement and the TDR-TT-TG measurement were good (ICC = 0.852, ICC = 0.864), and the corresponding value of TT-PCL measurement was also good (ICC = 0.758). The three measurements had good agreement among the different observers (ICC values > 0.75), and the TDR-TT-TG measurement has the best interobserver consistency (ICC = 0.864), which illustrates that the reliability of the three methods is superior.

**Table 1:** TT-TG versus TDR-TT-TG versus TT-PCL reliability (distance, mm, x̄ ± SD)

Parameter	Observer 1	Observer 2	Combined	ICC^a^
TT-TG on CT	19.0 ± 4.0	19.9 ± 4.2	19.5 ± 4.1	0.852
TDR-TT-TG on TDR	18.9 ± 4.1	19.1 ± 3.9	19.0 ± 4.0	0.864
TT-PCL on MRI	26.8 ± 4.9	24.8 ± 5.2	25.8 ± 5.1	0.758

*TT-TG* tibial tubercle–trochlear groove, *TDR-TT-TG* tibial tubercle-trochlear groove under three-dimensional reconstruction, *TDR* three-dimensional CT reconstruction, *TT-PCL* tibial tubercle-posterior cruciate ligament

^a^ICC > 0.75 was considered to represent good agreement

### Comparison of the three methods for assessing PFI

Compared with the control group, the values of TT-TG distance, TDR-TT-TG distance, and TT-PCL distance were obviously higher in PFI patients, and the difference was statistically significant (*P* = 0.000, *P* = 0.000, *P* = 0.000). There was no statistically significant difference between the TT-TG distance and the TDR-TT-TG distance, we found no significant difference. The TT-PCL distance was larger than both the TT-TG distance and the TDR-TT-TG distance; meanwhile, the difference was statistically significant (Table 2).

**Table 2:** Comparison of three sets of data measurements (*n* = 47, mm)

Parameter	Patient group	Control group	*t* value	*P* value
TT-TG on CT	19.0 ± 3.8	14.7 ± 2.4	6.594	0.000
TDR-TT-TG on TDR	19.0 ± 3.7	14.3 ± 2.5	7.226	0.000
TT-PCL on MRI	25.1 ± 3.6	21.5 ± 3.4	5.010	0.000
*t* value	− 0.069	− 7.958	− 7.958	
*P* value	0.945^a^	0.000^b^	0.000^c^	

Statistically significant difference (*P* < 0.05)

*TT-TG* tibial tubercle–trochlear groove, *TDR-TT-TG* tibial tubercle-trochlear groove under three-dimensional reconstruction, *TDR* three-dimensional CT reconstruction, *TT-PCL* tibial tubercle-posterior cruciate ligament

^a^TT-TG VS TDR-TT-TG

^b^TT-TG VS TT-PCL

^c^TDR-TT-TG VS TT-PCL

### Bland-Altman analysis of TT-TG and TDR-TT-TG in patients with PFI

**Fig. 2:**
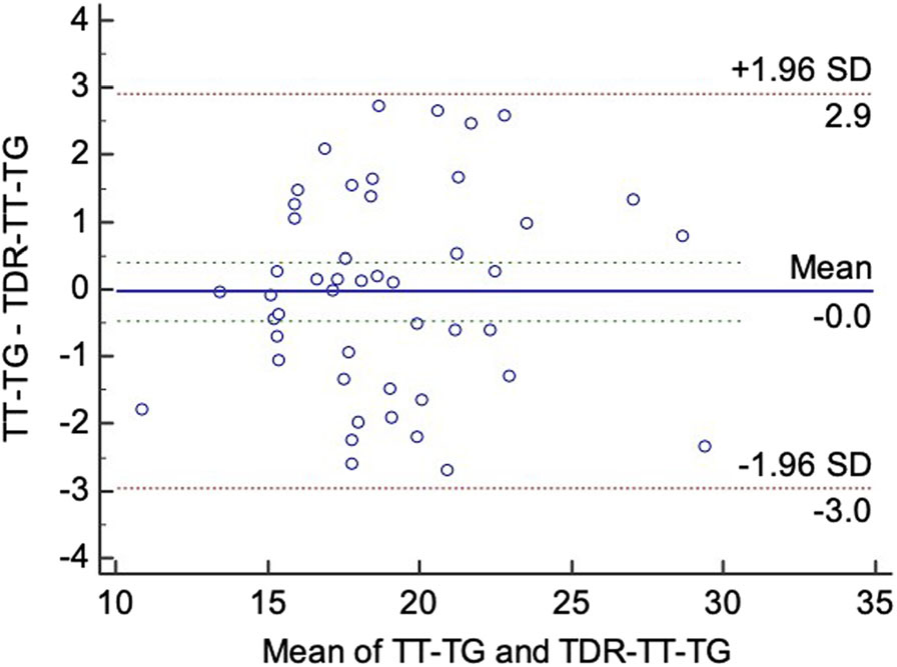
Bland-Altman analysis results of the TT-TG and TDR-TT-TG distances in patients with PI. TT-TG, tibial tubercle-trochlear groove; TDR-TT-TG, tibial tubercle-trochlear groove under three-dimensional reconstruction

Bland-Altman analysis of the two measurement methods showed that the mean difference between the TT-TG and TDR-TT-TG distances was *d* = 0.04 mm, and the 95% limit of agreement (95% LOA) was − 2.71 to 2.78 mm. Figure 2 demonstrates that all the points were within the LOA; the absolute value of the difference measured by the two methods was at most 2.73 mm; moreover, the amplitude of the phase difference was clinically acceptable. Therefore, the bias of the two measurement methods was slight, and the consistency was good, indicating that the two methods can be replaced by each other.

## Discussion

In this study, we used a new method based on a TDR modeling to measure TT-TG distance. The TDR-TT-TG method is only required for the completion of measurement in one layer, and our results illustrated that it has a higher interobserver consistency (ICC = 0.852) than the other two methods in our study. Furthermore, the Bland-Altman analysis indicated that the data measured by the conventional CT and TDR had good consistency, and the bias was slight; thus, the two methods are interchangeable. These results demonstrated that the TDR measurement is a reliable and feasible method. Similarly, recent research by Nha et al. [27] also illustrated that the TDR method has the advantages of smaller inter-rater variability, lower measurement bias, better reliability, and even be suitable for those patients with severe trochlear dysplasia. In their study, they established the trochlear line by connecting two deepest points of trochlear groove and the tibial tubercle line by connecting two most obvious markers, then drew a horizontal line through the midpoint of an imaginary patella to the tuberosity line as TT-TG distance. As their method requires more procedures, hence, we built the trochlear line through the deepest points of trochlear groove and the tuberosity line through the most recognizable point of tibial tuberosity. Moreover, they adjusted patients’ knee in a flexion with 30°. However, we thought in practice that it is difficult to ensure the consistency of the patients’ flexion angle and that excess flexion of knee may lead to false-negative results [6]. So we chose to have the knee fully extended for examination. Nevertheless, more well-designed studies are needed to confirm which measurement is superior. In any case, TDR images can intuitively and comprehensively display the bony structure of the knee joint, which can indicate abnormal development of the patella and femoral trochlea as well as excessive abnormalities of the tibial tuberosity.

The TT-TG distance we measured was relatively close to that of Anley et al. [19] (TT-TG 17.72 ± 5.15 mm). Additionally, in their study, the ICC of the TT-TG distance on CT was 0.97, with a very high reliability. And their findings have also proved that the measurements for the TT-TG cannot be used interchangeably between CT and MRI, and currently accepted values for TT-TG are based on CT scans [19]. The latest review [20], which took into account the effects of different populations, confirmed that the mean TT-TG distance measured on CT for patients with PFI was 18.33 mm, while the corresponding value was 12.85 mm for the control group, and they defined a new threshold of 15.5 ± 1.5 mm for TT-TG distance qualified on CT, Our measurement results are different from that of this review both in patients with or without PFI. In the study of Prakash et al. [28], the mean TT-TG distance in patients with PFI was 19.05 ± 4.8 mm, and the value is very close to ours; meanwhile, they have assessed many factors like knee rotation angle, femoral anteversion, trochlear groove medialization, and tibial torsion. Finally, they drew a conclusion that except for tuberosity lateralization, knee rotation was an important factor affecting the TT-TG distance. In addition, the TT-TG distance may be affected by ethnicity [29]. So we speculate that these differences are related to the factors mentioned above.

We found that the mean TT-PCL distance of PFI patients was 25.1 ± 3.6 mm, which was larger than the TT-PCL distance in the other studies [19, 30, 31]. That might be due to different MRI scanner, knee coils, and MRI protocols or due to ethnicity with for example differences in femoral and tibial rotations. As we thought, the data of three studies [19, 30, 31] above are all from Caucasians, and there was no indication of the race to which the data applied. A study of 566 children with PFI from the USA found that the average TT-PCL distance was 19.9 mm in normal children and 21 mm in children with PFI [32], and their research also found that the TT-PCL distance increased with age in the pediatric population. However, Boutris et al. [24] concluded that the TT-PCL threshold was identified as 21 mm in adults. And combined with the research results of Seitlinger et al. [23], the average TT-PCL distance was 26 ± 1.8 mm in adults with PFI, and they concluded the pathologic TT-PCL threshold was 24 mm. Therefore, we believe that the determination of the TT-PCL pathological threshold needs further research.

## Limitations

However, our study has some limitations. We ensured that the knee joint was in a 0° flexion position during CT examination, but knee coils for MRI testing we used could result in the knee being positioned in varus with slight knee flexion. Furthermore, although we tried to keep the knee as straight as possible during the examination, the angle of knee flexion may be different due to the different sizes of the limbs. Compared to TT-PCL, the measurement of TDR-TT-TG has a certain amount of radiation in the CT examination and a certain subjectivity in the selection of the lowest point of the femoral trochlea and the most cephalic point of the tibial tuberosity. While the consistency between observers was good, the clinical application of the TDR-TT-TG distance requires more studies.

## Conclusion

The TDR imaging technique for the measurement of the TT-TG distance has superior operability and better interobserver consistency. It may be an ideal alternative to the conventional TT-TG distance measurement.

## Data Availability

All data are included in the manuscript.

## Abbreviations

CT: Computed tomography
DSDECT: Dual-source dual-energy computed tomography
FOV: Field of view
ICC: Intraclass correlation coefficient
LOA: Limits of agreement
MRI: Magnetic resonance imaging
PFI: Patellofemoral instability
PCL: Posterior cruciate ligament
PDW-SPAIR: Proton density-weighted spectral attenuated inversion recovery
PDW-SPIR: Proton density-weighted spectral inversion recovery
TT-TG: Tibial tubercle to trochlear groove
TT-PCL: Tibial tubercle-posterior cruciate ligament
TDR: Three-dimensional reconstruction
TDR-TT-TG: TT-TG distance under three-dimensional CT reconstruction
TR: Repetition time millisecond
TE: Echo time millisecond
TSE: Turbo spin-echo
